# Non-Destructive Thickness Measurement of Energy Storage Electrodes via Terahertz Technology

**DOI:** 10.3390/s25133917

**Published:** 2025-06-23

**Authors:** Zhengxian Gao, Xiaoqing Jia, Jin Wang, Zhijun Zhou, Jianyong Wang, Dongshan Wei, Xuecou Tu, Lin Kang, Jian Chen, Dengzhi Chen, Peiheng Wu

**Affiliations:** 1 Research Institute of Superconductor Electronics, School of Electronic Science and Engineering, Nanjing University, Nanjing 210023, China; gaozhengxian@sina.com (Z.G.); tuxuecou@nju.edu.cn (X.T.); kanglin@nju.edu.cn (L.K.); chenj63@nju.edu.cn (J.C.); phwu@nju.edu.cn (P.W.); 2 Kexin Communication Technologies Co., Ltd., Shenzhen 518116, China; gczero@163.com (J.W.); wisechow@163.com (Z.Z.); wangjianyong118@sina.com (J.W.); chdzh@szkexin.com.cn (D.C.); 3 Purple Mountain Laboratory, Nanjing 211111, China; 4 Shenzhen Institutes of Advanced Technology, Chinese Academy of Sciences, Shenzhen 518055, China; ds.wei@siat.ac.cn

**Keywords:** terahertz time-domain spectroscopy (THz-TDS), energy storage electrodes, non-destructive testing, lithium iron phosphate (LFP) batteries, sinc wavelet threshold

## Abstract

Precision thickness control in new energy electrode coatings is a critical determinant of battery performance characteristics. This study presents a non-destructive inspection methodology employing terahertz time-domain spectroscopy (THz-TDS) to achieve high-precision coating thickness measurement in lithium iron phosphate (LFP) battery manufacturing. Industrial THz-TDS systems mostly adopt fixed threshold filtering or Fourier filtering, making it disssssfficult to balance noise suppression and signal fidelity. The developed approach integrates three key technological advancements. Firstly, the refractive index of the material is determined through multi-peak amplitude analysis, achieving an error rate control within 1%. Secondly, a hybrid signal processing algorithm is applied, combining an optimized Savitzky–Golay filter for high-frequency noise suppression with an enhanced sinc function wavelet threshold technique for signal fidelity improvement. Thirdly, the time-of-flight method enables real-time online measurement of coating thickness under atmospheric conditions. Experimental validation demonstrates effective thickness measurement across a 35–425 μm range, achieving a 17.62% range extension and a 2.13% improvement in accuracy compared to conventional non-filtered methods. The integrated system offers a robust quality control solution for next-generation battery production lines.

## 1. Introduction

Electrode coating is a key process in the production of energy storage products such as lithium iron phosphate (LFP) batteries and supercapacitors [1]. The coating speed can reach 15 m per minute, and more than 95% of defective products are caused by uneven thickness. Ultrasonic thickness gauging is prone to acoustic impedance and mismatch errors (≥3% for LiFePO_4_–electrolyte interfaces) and requires couplant media [2]. Laser Doppler vibrometry is limited by surface roughness (Rq > 1 μm causes speckle noise) and a low scanning speed (<1 frame/s) [3]. Optical interferometry is affected by refractive index dispersion (Δn/n > 5% across 400–700 nm) and requires transparent substrates [4]. X-ray fluorescence (XRF) suffers from concerns about radiation safety [5]. These issues make it difficult to achieve real-time online non-destructive measurement in industrial inspection of energy storage electrodes. The unique characteristics of terahertz time-domain spectroscopy (THz TDS), such as transient, low-energy, non-ionizing radiation and high precision [6], make it promising for application in non-destructive testing. The THz coating thickness measurement technology is mainly based on time-of-flight detection, theoretical modeling, and optimization algorithm processing [7].

In recent years, several studies have shown the potential of terahertz technology for coating thickness measurements. In 2007, J. Zeitler et al. first applied THz-TDS to offline coating thickness characterization [8]. In 2009, White et al. used a reflective system to measure the thickness of thermal barrier coatings. The detected thickness ranges from 136 to 185 μm [9]. In 2011, Robert et al. proposed real-time THz monitoring for manufacturing processes as an online application [10]. In 2013, Hussain et al. proposed a theoretical model for the thickness and refractive index of five materials (fused silica, glass, silicon, Teflon, and Perspex), with an accuracy deviation of 6 to 18 μm [11]. In 2016, Krimi et al. measured the thickness of automotive coatings using the stochastic differential evolution (DE) algorithm. Although the accuracy was less than 5 μm, the calculation complexity hindered real-time detection [12]. In 2019, Tu et al. preprocessed signals using wavelet analysis, with different thickness data (45–127 μm) and an RMSE (root mean square error of measured and standard thickness) of 0.0036 [13]. In 2022, Zhang et al. utilized differential evolution and empirical mode decomposition (EMD) reconstruction algorithms, with a base thickness limit of 40 μm [14]. In the same year, Sun et al. adopted the long short-term memory (LSTM) algorithm to enable the detection of local extreme (THz) peaks, with the single-point detection time ranging from 0.17 to 0.26 s [15]. In 2023, Gao et al. examined a supercapacitor coating with a thickness of 65 μm using an optimized time of flight (TOF) and the THz-TDS system [16]. In 2023, Cao et al. improved deep learning speed with parsing models combined with a convolutional neural network and a gated recurrent unit (CNN-GRU), with different thickness data (200–400 μm) [17].

Terahertz technology holds significant commercial promise for measuring coating thickness in laboratory settings. Nevertheless, the majority of current research efforts are centered around coatings, such as paints, plastics, and ceramics [18]. Moreover, these studies are typically conducted in dry environments or those filled with nitrogen, rendering them unsuitable for practical factory-level production scenarios.

In this study, we designed a highly integrated, distributed fiber-optic TDS thickness measurement system. A key feature of this system is the separation of the detection probe from the spectrometer. We utilized the Savitzky–Golay (SG) and wavelet threshold filtering algorithms to optimize the measurement data. These algorithms effectively mitigated noise and broadened the thickness measurement range. Additionally, by leveraging an amplitude-based algorithm to calculate the refractive index, we eliminated the requirement for supplementary equipment. This breakthrough enables real-time analysis and processing of thickness-related information directly on the electrode coating production line.

Using a lithium iron phosphate (LFP) coating as a model example, we conducted a comprehensive spectral analysis of samples with thicknesses ranging from 35 to 425 μm. The obtained results were then seamlessly integrated into the software interface, enabling real-time online thickness measurement. This advancement significantly streamlines industrial quality control procedures.

## 2. Materials and Methods

**Figure 1:**
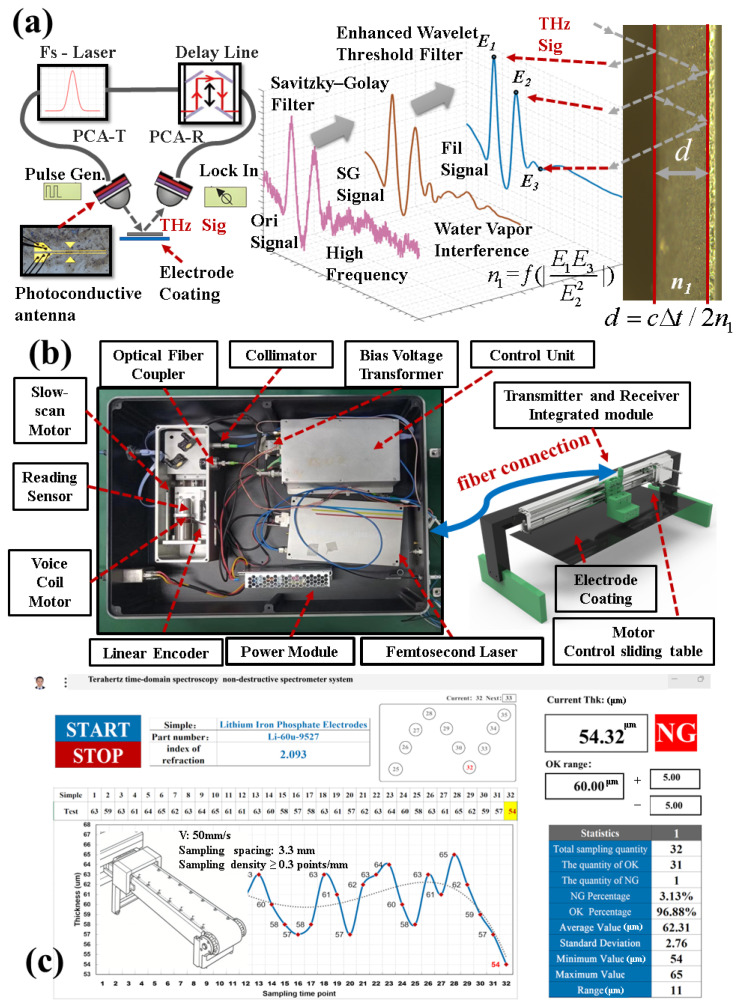
Terahertz industrial non-destructive testing (NDT) thickness measurement system. (**a**) Schematic diagram of THz thickness measurement system and its filtering algorithm. (**b**) Online measurement system, all-fiber THz-TDS, and its components. (**c**) Real-time online thickness measurement monitoring and analysis interface.

Figure 1a shows the process of terahertz signal generation, filtering, refractive index determination, and thickness measurement. For terahertz signal generation, a femtosecond laser pulse separates the pumping and detection paths. The pump laser, combined with a pulse generator (Pulse Gen), excites a photoconductive antenna (PCA-T) to generate a terahertz signal. After passing through the test sample, the reflected signal, carrying spectral information, is received by the detection antenna (PCA-R). The phase locker and delay line are crucial for receiving and comparing spectral signals.

In the filtering stage, the original air state (Ori) waveform has numerous burrs and high-frequency noises. A Savitzky–Golay (SG) filter is used to obtain a relatively smooth SG signal. However, the SG signal still has interference from water vapor scattering and transmission. Thus, a wavelet threshold filtering (Fil) method with the sinc function as the segmental threshold parameter is adopted to obtain the Fil signal. This Fil signal has a higher signal-to-noise ratio and resolution, aiding the extraction of key spectral peaks.

The approximate refractive index is calculated from the amplitude coefficients of multiple terahertz reflections [19]. When choosing sample materials, those with strong reflection peak signals and an obvious third reflection peak are preferred. Using the amplitudes of multiple reflection peaks to derive the approximate refractive index simplifies testing and reduces the need for other instruments.

The time-of-flight (TOF) method [20] is used for thickness measurement. Calculations follow the formula $d=c\Delta t/2n_{1}$, where d is the thickness, c is the speed of light, Δt is the flight time, and $n_{1}$ is the refractive index. These methods enable effective measurement of electrode coating thickness-related parameters.

To rapidly acquire high-resolution terahertz spectral signals and enable the miniaturized and distributed deployment of a fiber-optic THz-TDS thickness measurement system, we enhanced the conventional terahertz time-domain spectrometer [21]. As depicted in Figure 1b, fiber coupling and dispersion compensation techniques were used to replace the optical loop, resulting in an all-fiber connection.

The system incorporates a femtosecond laser [22] with the following parameters: a wavelength of 1560 nm, a repetition frequency of 100 MHz, a pulse width as brief as 58 fs, and a maximum power of 175 mW. This femtosecond laser serves to generate terahertz waves, and the associated spectral system features a time resolution of 0.1 ps. Simultaneously, the optical delay line structure was miniaturized by implementing a voice coil motor and grating scale. The optical fiber is extended to the motor detection stage, and the receiving and transmitting modules of the photoconductive antenna are integrated [23] (1.2 THz bandwidth, average optical power of 1–15 mW, and 4.5 nA peak photocurrent).

The spectrometer system control unit, equipped with a 1 GHz DSP, coordinates the operation of various components and can complete one or more acquisitions [24]. The power supply ripple was less than 10 mV, and the bias voltage stability was ±0.3%. The motorized displacement stage has a travel range of 100 cm, a precision resolution of up to 1 μm, an angular resolution of 0.01°, and the distance between the photoconductive antenna probe and the coating is 1–3 cm. A communication link was established to ensure synergy.

As shown in Figure 1c, the THz measurement speed is 15 waveforms per second (with a 66 ms interval). For an industrial conveyor running at v = 50 mm/s, the spacing between adjacent measurement points is calculated as v × 66 ms = 3.3 mm. To validate the rationality of this spacing, a 200 mm × 200 mm electrode was scanned at 1 mm intervals. The results show that the standard deviation of thickness fluctuations increased from 0.7 μm to 1.2 μm (still within the industrial requirement of ±5 μm), ultimately achieving industrial-grade online monitoring with a sampling density ≥ 0.3 points/mm.

The software interface is integrated into the electrode coating thickness measurement system. This integration empowers the system to conduct real-time line-scan thickness measurement, issue non-good (NG) alarms, and offer batch information feedback. Regarding the spectrometer system, its dimensions are 520 × 400 × 140 mm^3^, and it weighs 20 kg. In comparison to the mainstream Tera Sys-AIO terahertz time-domain spectrometer, which weighs 30 kg and has approximate dimensions of 550 × 450 × 280 mm^3^, our system is approximately 50% smaller and 30% lighter. Other parameters are presented in Table 1.

## 3. Results and Discussion

### 3.1. Combined SG and Enhanced Wavelet Thresholding Algorithms

We initiated our signal processing process with the Savitzky–Golay filter [25]. For signal smoothing and fitting, we applied polynomial fitting and smoothing, based on the least-squares method, to the data within the sliding window. A second-order polynomial (Polynomial = 2) was selected, and the length of the sliding window was set to 25 (Frame Len = 25). This was carried out to guarantee that the peak position and peak height of the spectral peak signal remained undamaged during the noise removal process.

**Figure 2:**
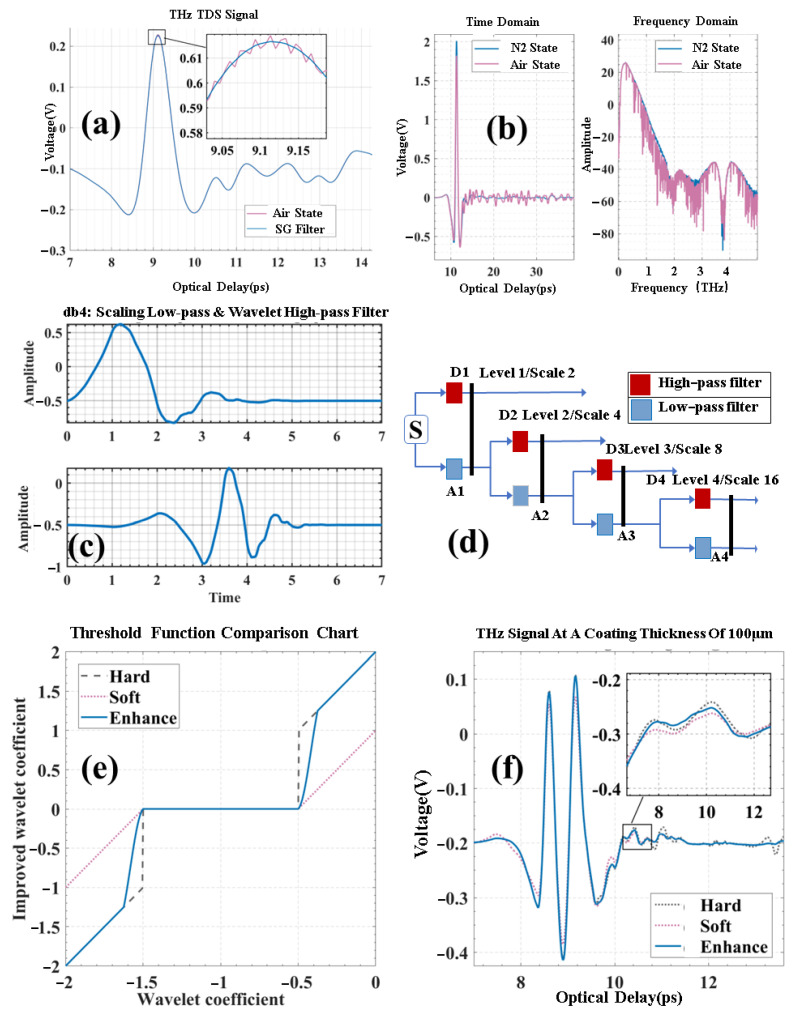
The characteristic plots of high-frequency signal filtering and enhanced wavelet threshold filtering. (**a**) The plot of the Savitzky–Golay filter. (**b**) The time-domain and frequency-domain spectra of terahertz detection signals with noise under different air states. (**c**) The scaling low-pass and wavelet high-pass filters for the scaling function of the DB wavelet basis function. (**d**) The schematic diagram of the signal under wavelet decomposition conditions. (**e**) The enhanced segmented thresholding method, along with a comparison of the soft and hard thresholding functions. (**f**) The comparison diagram of the filtering effect of the enhanced wavelet thresholding.

As shown in Figure 2a, the filtered signal appears flat. But in the 0.1–3 THz processed signal, the time- and frequency-domain signals in Figure 2b still contain significant noise. Analysis reveals that this noise mainly stems from terahertz wave scattering and reflection due to airborne water vapor [26]. These noise signals, with time-frequency localization and non-stationary traits [27], are a mix of various frequencies.

Normally, non-polar gases like dry air or nitrogen are used to reduce water vapor noise on terahertz radiation. However, in manufacturing sites, space and environmental constraints rule out nitrogen use. Thus, we propose an improved wavelet thresholding algorithm for optimal filtering. (All measurements are carried out under conditions of humidity ≤ 30% RH to further explore the humidity dynamic compensation algorithm).

Firstly, considering the length of the input signal, which is 8016, the Daubechies 4 (‘db4’) wavelet type is selected. Its waveform is shown in Figure 2c. The wavelet undergoes filtering via the scaling low-pass function and the mother wavelet function to carry out the denoising process with time-frequency localization characteristics. By taking advantage of the low-pass filtering property of the scaling function and the high-pass filtering property of the mother wavelet function, the denoising can be localized in both the time and frequency domains. Moreover, the symmetry of the wavelet is utilized to preserve the signal’s shape [28].

After performing the wavelet transform, a series of wavelet coefficients is obtained. The decomposition process, depicted in Figure 2d, involves using special low-pass and high-pass filters to filter the signal, discarding half of the samples post-filtering, and reconstructing the sub-bands. Here, we opt for eight decomposition layers, which ensures an effective filtering effect while maintaining computational efficiency.

From the time and frequency domains of the acquired signals in Figure 2b, it is evident that the noise is widely distributed. In this study, the VisuShrink threshold is applied [29], which is expressed as follows: σ represents the standard deviation of the noise, and *N* is the signal length. The energy of a terahertz time-domain signal is mainly concentrated in the larger wavelet coefficients, whereas the noise is uniformly distributed among all wavelet coefficients. Thus, by calculating the median of the absolute values of the wavelet coefficients, a relatively stable value can be obtained to characterize the noise level. Using the MAD (median absolute deviation) method [30], let $C=\left\{c1,c2,\ldots ,cN\right\}$ be the set of coefficients after the wavelet transform. Then, absolute values are computed to determine the median.

(1) $$\sigma =\frac{median(|C|)}{0.6745}$$

Hard thresholding sets the portion of the signal with absolute values below the threshold to zero. This method is straightforward yet somewhat rudimentary. Although it preserves certain signal characteristics, it can result in signal discontinuities. In contrast, soft thresholding reduces the magnitude of the part of the signal with an absolute value greater than the threshold. This yields a smoother signal and mitigates the impact of noise, but it may lead to the loss of some signal details.

To tackle the limitations of traditional threshold functions, the Garrote threshold function [31] incorporates the concept of an asymptote to alleviate the bias caused by the soft threshold function. Nevertheless, its convergence to the true value is slow, and it fails to completely eliminate the bias.

In our research, we utilized the sinc function, which is continuous and has a rapidly decaying main lobe. This allows the function to approach the actual value more swiftly in the transition region and minimizes the loss of valuable information during the denoising process. As depicted in Figure 2e, by integrating the sinc function with the hard thresholding function, information is fully preserved after passing through the transition region. This resolves the issues associated with the wavelet thresholding function and achieves a superior denoising effect compared to soft and hard thresholds alone. In this study, we propose a multi-layer wavelet threshold function based on the sinc function and refine the threshold function as follows:

(2) $$W=\left\{\begin{array}{ll} W_{j,k}, & |W_{j,k}|\geq \lambda_{2}\\ W_{j,k}-\lambda_{1}{\left[sinc\alpha (W_{j,k}-\lambda_{1})\right]}^{2}, & \lambda_{2}>W_{j,k}\geq \lambda_{1}\\ 0, & |W_{j,k}|<\lambda_{1}\\ W_{j,k}+\lambda_{1}{\left[sinc\alpha (W_{j,k}+\lambda_{1})\right]}^{2}, & -\lambda_{2}<W_{j,k}\leq -\lambda_{1} \end{array}\right.$$

Here, under the first high-frequency coefficient, there is a decomposition scale. Let W represent the processed wavelet coefficients, α be the adjustment parameter, and $\lambda_{1}$ be the selected threshold. $\lambda_{2}$ is the transition-end threshold, with a value of $\lambda_{2}=\lambda_{1}+\alpha^{-1}$, where α s the adjustment parameter. When α→0, the transition region is short, and the function approximates the hard threshold function. When α→+∞, the transition region is long, and the function approximates the soft threshold function.

When the peak amplitude is relatively large, the algorithm close to the hard threshold can retain more feature signals. After leaving the third peak, the filtering effect is enhanced to reduce the impacts caused by water vapor. From Figure 2f, the enhanced threshold function can achieve the best balance between filtering and the retention of feature values.

Table 2 compares the effects of different thresholds. The enhanced threshold filtering has outstanding advantages. Its signal-to-noise ratio (SNR) is as high as 18.181, filtering out 19.69% more noise than other algorithms. This ensures pure and clear filtered signals, providing reliable support for subsequent analysis. The mean squared error (MSE) is only 0.000107, and the root mean squared error (RMSE) is 0.010, reducing the filtering error by 50.55% compared to other methods, allowing for accurate signal restoration, even in demanding scenarios such as scientific research and precision measurements. The normalized cross-correlation (NCC) reaches 0.992, an increase of 0.29%, indicating that key information is retained and ensuring that the filtered signals can be transmitted and applied as originally intended to convey the value.

The technology combines high computational efficiency (suitable for real-time online processing compatible with 15 waveforms/s measurement speed) and intuitive physical interpretation (peak positions directly correlate with coating interface reflections, facilitating engineering deployment) but has limitations, including sensitivity to system spectral responses (e.g., frequency-dependent THz source attenuation), errors in thick coatings (>100 μm, ~5% error) due to neglecting refractive index dispersion, and ripple noise introduced via water vapor absorption (mitigated by spectral filtering). A comparison with full-spectrum fitting algorithms shows that peak analysis is suitable for rapid production-line screening, while full-spectrum fitting is preferable for laboratory-grade precision.

By using a standard silicon wafer for impulse response calibration, applying deconvolution processing to suppress water vapor ripple noise, and introducing the Drude–Lorentz model [32] to fit frequency-dependent refractive indices and account for material dispersion characteristics, the noise amplitude and thickness error in related measurements are significantly reduced. Future projects may consider further optimizing the calibration process, exploring more adaptable models to address complex environmental factors, or integrating other advanced algorithms to achieve continuous enhancement of measurement performance.

### 3.2. Software Calculation of Refractive Index at Specific Thickness Amplitudes

**Figure 3:**
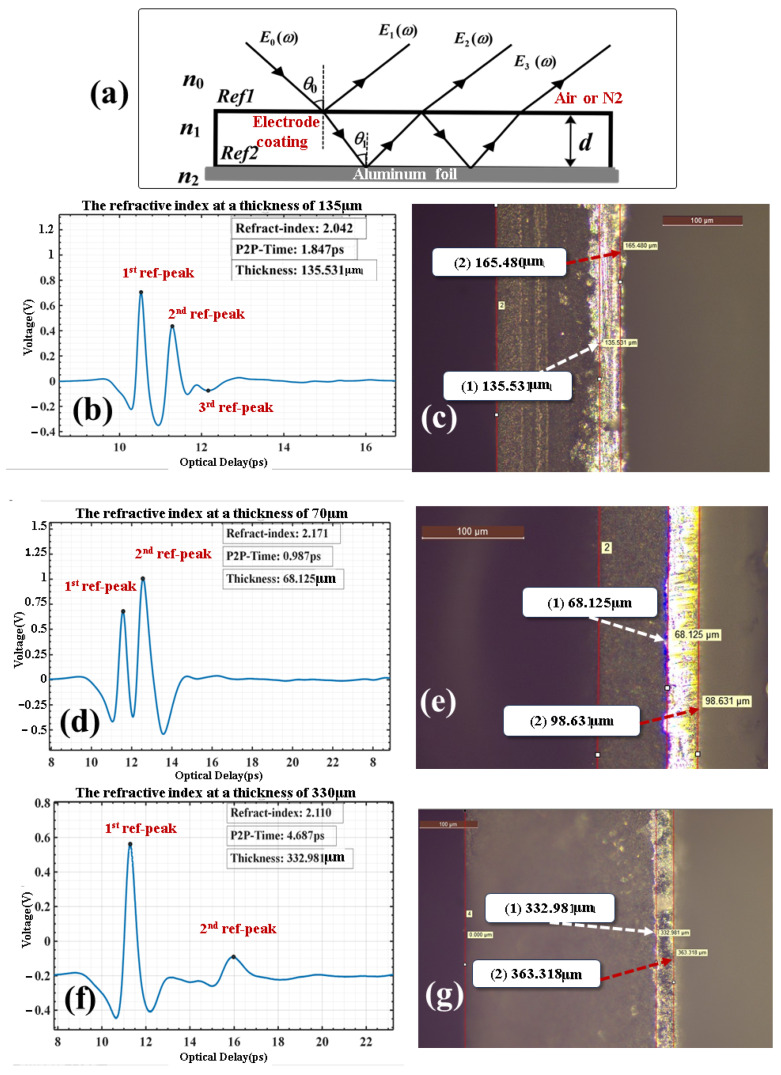
(**a**) A schematic diagram of the multiple transmission and reflection of a terahertz wave incident from the coating to the electrode coating and the aluminum foil. Here, Ref1 represents the first reflection surface of the electrode coating, and Ref2 represents the second reflection surface between the aluminum foil and the electrode coating. (**b**) Terahertz wave signal maps of a 135 μm thick coating under air and nitrogen environments. (**c**) Metallurgical microscopy comparison images of a 135 μm thick coating. (**d**) Terahertz wave signal map of a 70 μm thick coating under a nitrogen environment. (**e**) Metallurgical microscopy comparison images of a 70 μm thick coating. (**f**) Terahertz wave signal map of a 330 μm thick coating under a nitrogen environment. (**g**) Metallurgical microscopy comparison images of a 330 μm thick coating.

When a terahertz wave travels through a multi-layer medium, the intensity and time delay of the reflected wave hinge on the refractive index and thickness of each medium layer. As illustrated in Figure 3a, the terahertz wave experiences multiple transmissions and reflections between the electrode coating and the aluminum foil. Precisely, Ref1 denotes the first reflection surface of the electrode coating, while Ref2 represents the second reflection surface at the interface between the aluminum foil and the electrode coating.

Based on Fresnel’s formulas, the reflection and refraction coefficients are associated with the refractive index of the medium. The experimental incident angle of the THz beam was θ_0_ = 0.5°. Fresnel reflection theory was used in modeling, with the propagation time corrected as *t* = 2*d*·*n*/*c*·cosθ_0_. Simulations comparing 0° and 0.5° incident angles showed that the thickness error between the two cases was only 0.04%. To simplify calculations, the incident angle and reflection angle in this experiment were approximated as 0°. The Kirchhoff method is applied. This method modifies the incident terahertz wave into a P-wave [33]. The frequency domain of each reflection peak of the THz signal, along with the attenuation within one internal reflection distance, is presented in Equation (3).

(3) $$E_{n}(\omega )=\left\{\begin{array}{ll} r_{01}E_{0}(\omega ) & n=1\\ t_{01}{\left(r_{12}\right)}^{n-1}{\left(r_{10}\right)}^{n-2}\;t_{10}\;e^{i(n-1)\beta }\;E_{0}(\omega ) & n>1 \end{array}\right.$$

Let $E_{0}(\omega )$ denote the first reflected signal and $E_{n}(\omega )$ represent the frequency-domain representation of the nth reflection peak. The phase factor c is the speed of light, and ω is the angular velocity. When the wave is incident from the air layer onto the electrode coating, the reflectance and transmittance are denoted as $r_{01}$ and $t_{01}$, respectively. Conversely, when the wave is incident from the electrode coating onto the air layer, the reflectance and transmittance are $r_{10}$ and $t_{10}$, respectively. Moreover, $r_{12}$ is the reflectivity of the aluminum foil layer when the wave is incident from the electrode coating. In the case of total reflection, it is approximated as 1.

According to Rouard’s equivalent-interface theory [34], in the process leading up to the third reflection, the optical path features a light-dense medium incident on a light-rare medium. Consequently, the third reflection peak is negative. The elimination of $e^{i(n-1)\beta }$ can be accomplished by calculating the ratio of the reflection peak frequency domains one to three times using Equation (4).

(4) $$P=\frac{E_{1}E_{3}}{E_{2}^{2}}=\frac{r_{01}r_{10}}{t_{01}t_{10}}$$

Based on the Kirchhoff approximation [35], the correction $\;E_{suf}=E_{mir}e^{-{\left(4\pi \delta /\lambda \right)}^{2}}$ is applied. Let $s=e^{-{\left(4\pi \delta /\lambda \right)}^{2}}$, which quantifies the impact of terahertz wave attenuation. This attenuation is caused by factors like the surface roughness of the electrode coating, the molecular structure of the electrode layer, and material absorption. The value of s ranges between 0 and 1. Subsequently, the following formula can be derived by combining the Fresnel formula and (Equation (4)).

(5) $$|P|=\frac{{\left(n_{1}-1\right)}^{2}}{4n_{1}s}$$

In the studied LPF, *s* = 0.5 serves as a calibration value. When applied, the refractive indices calculated via relevant formulas show optimal agreement with high-precision optical measurements (e.g., spectroscopic ellipsometry under identical conditions). This not only enhances the consistency between theoretical predictions (based on Kirchhoff approximation and Fresnel equations) and experimental data but also reflects the material’s typical attenuation behavior.

Note: The *s* = 0.5 calibration is specific to LFP under the reported conditions. For other materials, s must be re-determined using methods like spectroscopic ellipsometry due to variations in surface roughness, molecular structure, and absorption, as direct extrapolation is invalid.

For the lithium iron phosphate (LFP) hybrid material, as $n_{1}>1$, $n_{1}$ takes the positive sign in the corresponding equation, the Equation is as follows.

(6) $$n_{1}=\left(|P|+1\right)+\sqrt{\left|P{|}^{2}+2|P\right|}$$

When comparing multiple samples with a thickness of 135 μm, the third reflection peak is negative. This occurs because the wave propagates from the aluminum foil (optically denser medium) to the LFP coating (optically rarer medium). In Figure 3b, the curve of the 135 μm sample in air is $E_{1}=0.632$, $E_{2}=0.439$, and $E_{3}=-0.079$. Substituting these values into Equation (4) gives |P|=0.259, and substituting them into Equation (6) yields $n_{1}=2.025$.

A 1000x-magnified Leica DM6000M metallurgical microscope (Leica Microsystems, Wetzlar, Germany) was used to observe 100 μm cross-sections, and the thicknesses of 70 μm (Figure 3e), 135 μm (Figure 3c), and 330 μm (Figure 3g) samples were measured as $Thk_{70\mu m}=68.125\;\mu m$, $Thk_{135\mu m}=135.531\;\mu m$, and $Thk_{330\mu m}=332.981\;\mu m$, with refractive indices of $n_{1-70\mu m}=2.171$, $n_{1-135\mu m}=2.042$, and $n_{1-330\mu m}=2.110$.

Table 3 presents refractive index deviation rates ranging from 0.88% to 4.73%, which are higher for thinner materials. The deviation of less than 5% calculated using the amplitude validates this method, reducing observation time and decreasing the dependence on microscopes.

Considering the higher SNR of the third reflection amplitude at a thickness of 135 μm, this characteristic thickness was exclusively selected for refractive index inversion. The measured results show high consistency with instrument calibration values, with deviations controlled within a minimal range. The third reflection signals at the other two thicknesses were below the detection threshold, making reliable signal acquisition and analysis unfeasible. For precise refractive index characterization of samples with different thicknesses, auxiliary optical measurement techniques such as spectroscopic ellipsometry should be integrated.

### 3.3. Spectra and Analysis of LFP Coating Materials with Different Thicknesses

**Figure 4:**
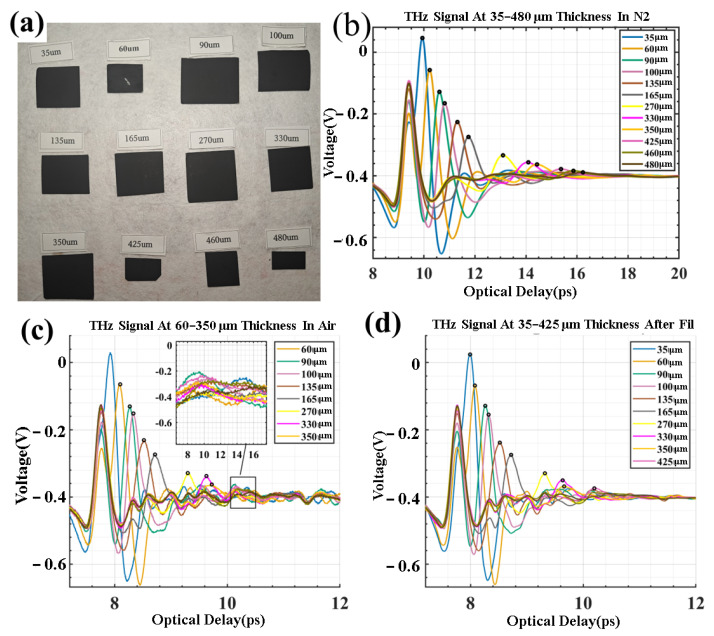
(**a**) LFP coating with a thickness ranging from 35 to 480 μm. (**b**) Terahertz signals of samples with a thickness from 60 to 350 μm under a nitrogen environment. (**c**) Terahertz signals of samples with a thickness from 60 to 350 μm in an air environment. (**d**) Terahertz signals of samples with a thickness from 35 to 425 μm under a thin-film condition. (The data are the average values of three repeated measurements, and the error bars are ±1σ).

By applying the aforementioned algorithms and programs, we conducted analyses of coatings with thicknesses ranging from 35 to 480 μm. Currently, these thicknesses are the most commonly used. The LFP coating materials were processed via the current mainstream carbon-coating procedure [36]. The main components of these materials include iron, phosphorus, lithium, carbon, conductive agents, binders, and finished products. As shown in Figure 4a, there are 12 groups of LFP coating materials.

We derived the sample thickness from terahertz signals and compared the outcomes with those measured using a metallurgical microscope, setting a maximum tolerance of 5%. As illustrated in Figure 4a, we fabricated 12 groups of LFP coatings with varying LFP coating thicknesses, spanning from 35 μm to 480 μm. The results were clearly observable in Figure 4b under a nitrogen atmosphere.

In contrast, when employing the traditional sampling method for detection in air, as shown in Figure 4c, the effective detection range was reduced to 60–350 μm. Such a limited range can pose an obstacle to the industrial application of actual electrode coatings.

Under the same air state, by applying S-filtering and enhanced wavelet threshold filtering to weak and overlapping peaks, as illustrated in Figure 4d, the detection range was expanded to 35–425 μm. This technological advancement allows for the accommodation of a wider variety of thicknesses, including thinner samples (35 μm) and thicker ones (425 μm). Moreover, the detection error can be kept within 3%. In practical industrial production, this approach shows a remarkable improvement in both accuracy and detection capacity compared to the traditional approach.

**Figure 5:**
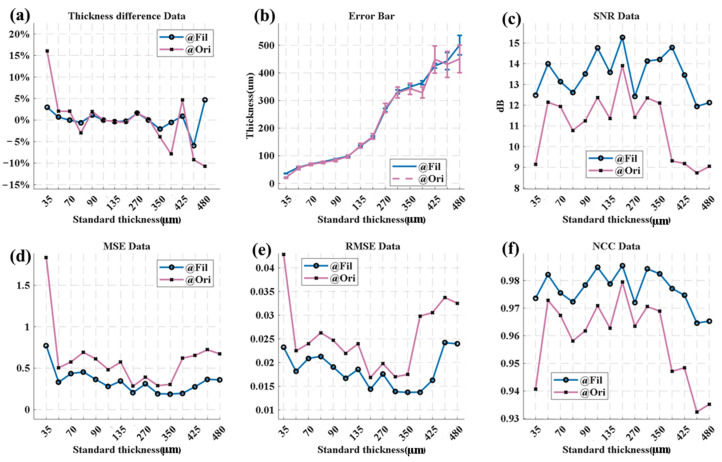
Statistics and analysis of thickness measurement data. (**a**) Thickness error rate (vs. nominal for electron microscopy) for samples of different thicknesses. (**b**) Error bar for multiple tests of samples, (**c**) Signal-to-noise ratio (SNR). (**d**) Mean square error (MSE). (**e**) Root mean square error (RMSE). (**f**) Normalized correlation coefficient (NCC). (The data are the average values of three repeated measurements, and the error bars are ±1σ).

As depicted in Figure 5, for the sample with a nominal thickness of 35 μm (the thinnest in Figure 5a), the thickness measurement error can be as high as 16.02%, highlighting a significant data discrepancy. When the thickness is within the range of 60 μm to 350 μm, the error can be kept within 5%. Yet, at 365 μm, the error increases to −7.90%. After applying wavelet threshold filtering, the thickness measurement error for samples with thicknesses from 35 μm to 425 μm is controlled between 1% and 3%, only rising to 5.91% at 460 μm.

The “Error Bar” graph in Figure 5b reveals that the filtered data aligns more closely with the nominal values and the measurements from the electron microscope and that the post-filtering error bars are shorter. Shorter error bars signify higher measurement precision. The maximum measurable thickness of the original detection scheme is 350 μm. For a sample with a nominal thickness of 365 μm, the test data are 335 ± 20 μm, and the deviation rate is 8%. However, after adopting the filtering scheme, the testable thickness can be extended to 425 μm. The signal-to-noise ratio (SNR) data at 365 μm in Figure 5c indicates that the SNR after filtering is 14.790, substantially higher than the initial SNR of 9.307 in the air-side data. This implies that wavelet threshold filtering reduces the interference from noise sources like water vapor.

In Figure 5d,e, at 365 μm, both the mean square error (MSE) and root mean square error (RMSE) of the filtered data are much smaller than those of the unfiltered data, suggesting that the filtered data are more representative of the actual thickness. The normalized correlation coefficient (NCC) value in Figure 5f shows that the filtered data exhibit better reliability and consistency compared to the pre-filtered data.

Overall, wavelet threshold filtering enhances the terahertz detection signals in terms of the SNR, RMSE, and NCC. Concerning the thickness error, the proportion of measurable data points after filtering is roughly 20% greater than that of the original signal, the RMSE is approximately 40% lower, the NCC is about 1–2% higher, and the SNR is around 2 dB–3 dB higher.

While this study focuses on energy storage electrodes (e.g., lithium iron phosphate), the proposed method based on wavelet denoising and peak amplitude refractive index measurement can be extended to other laminated dielectric materials (e.g., semiconductor films and polymer coatings) by adjusting material parameters (e.g., dielectric constant). Future work may explore applications in optical thin films or biomedical coatings.

## 4. Conclusions

The refractive index of the LFP material is computed through multiple amplitudes and the multi-peak amplitude relationship, with an error rate below 1% using THz-TDS measurement. This work represents a remarkable improvement in accuracy compared to the traditional method, which has an error rate between 5% and 10%. By utilizing the enhanced wavelet threshold filtering technique, the SNR increases by more than 30%. After implementing the algorithm, online, accurate, and real-time detection of electrode thicknesses ranging from 35 μm to 425 μm becomes feasible. The detection range is 17.62% broader than that of the traditional approach. According to the industry standard GB/T 31485-2015 [37], the thickness tolerance of energy storage electrodes is typically ±5%. The average thickness accuracy has been enhanced from ±3.51% to ±1.37%, which meets the needs of industrial-grade tolerances [38]. The detection time has been sharply reduced from the traditional 3 min of in-line stopping detection to just 5 s, covering the entire process, including data collection, filtering algorithm processing, numerical operation, user interface visualization, and result output. The THz-TDS-based detection method exhibits outstanding performance in terms of accuracy, efficiency, and environmental friendliness, holding great promise for industrial applications.

For future optimization in engineering application scenarios, high-precision auxiliary devices can be incorporated to minimize vibration, electromagnetic interference, and noise, providing protection against external disturbances. A comprehensive evaluation system for electrode coatings and a coating quality assessment method were established. Preliminary estimates indicated that the error in detecting air bubbles, gaps, and the moisture level of the coating is less than ±5%. A closed-loop feedback control system was built, capable of automatically adjusting the coating parameters within 1 s, ensuring that the error of the electrode’s thickness measurement is less than ±3 μm.

## 5. Patents

1. Zhengxian Gao, Xiaoqing Jia, Xuecou Tu, Peiheng Wu, and Jin Wang applied for a Chinese patent (No. CN202411485728.8) [39].
2. Zhengxian Gao, Xiaoqing Jia, Peiheng Wu, Jin Wang, Jianyong Wang, and Dongshan Wei applied for a Chinese patent (No. CN202411485764.4) [40].
3. Zhengxian Gao, Xiaoqing Jia, Xuecou Tu, Peiheng Wu, Zhijun Zhou, and Jin Wang applied for a Chinese patent (No. CN202411485766.3) [41].
4. Zhengxian Gao, Xiaoqing Jia, Xuecou Tu, Lin Kang, Jian Chen, Peiheng Wu, Zhijun Zhou, Jin Wang, and Jianyong Wang applied for a Chinese patent (No. CN202410415816.4). The remaining authors declare that they have no conflict of interest [42].

## Figures and Tables

**Table 1:** Core parameter table of terahertz spectrometer.

Performance Indicator	Effective Spectral Range	Dynamic Range	Time-Domain Length	Imaging Scanning Speed	Femtosecond Laser
Specification	0.1–4.0 THz	≥70 dB	53–300 ps	15 Hz	1560 nm

**Table 2:** SNR, MSE, RMSE, and NCC comparisons of different thresholds at 100 μm.

	SNR	MSE	RMSE	NCC
Hard threshold	16.777	0.000148	0.012	0.989
Soft threshold	15.190	0.000217	0.015	0.989
Enhance threshold	18.181	0.000107	0.010	0.992

The number of decimal places retained is adjusted according to the precision requirements of scheme comparison. Multiple decimal places for key parameters are used to reflect subtle differences.

**Table 3:** Analysis of materials with different thicknesses.

Sub-Fig	TOF (ps)	*n*_1_ (Microscope)	Thickness (μm) (Microscope)	Thickness (μm) (*n*_1_ = 2.025)	Thickness Deviation Rate
135 μm thick	1.847	2.042	135.531	136.721	0.88%
70 μm thick	0.987	2.171	68.125	71.350	4.73%
330 μm thick	0.746	2.110	332.981	338.922	1.78%

Thickness measurement: σ ≤ 1.5 μm over 10 positions (*n* = 10). Refractive index: σ ≤ 0.02 (*n* = 10).

## Data Availability

The data underlying the results presented in this paper are not publicly available at this time but may be obtained from the authors upon reasonable request.
